# Direct and In-Utero Exposure to Quaternary Ammonium Disinfectants Alters Sperm Parameters and mRNA Expression of Epigenetic Enzymes in the Testes of Male CD-1 Mice

**DOI:** 10.3390/toxics13090709

**Published:** 2025-08-23

**Authors:** Vanessa E. Melin, Terry C. Hrubec

**Affiliations:** 1 Department of Biomedical Sciences and Pathobiology, VA-MD College of Veterinary Medicine, Virginia Polytechnic Institute and State University, Blacksburg, VA 24061, USA; haasemeine.email@gmail.com; 2 Edward Via College of Osteopathic Medicine Virginia Campus, Blacksburg, VA 24060, USA

**Keywords:** quaternary ammonium compound, reproduction, epigenetics, fertility, sperm, testis, environmental contaminants

## Abstract

Quaternary ammonium compounds (QACs) are a class of chemicals used for their antimicrobial, surfactant, and antistatic properties. QACs are present in many consumer products, and people are regularly exposed to them. We have previously shown reproductive toxicity in mice exposed to the disinfectants alkyl dimethyl benzyl ammonium chloride (ADBAC) and dodecyl dimethyl ammonium chloride (DDAC). To assess the long-term reproductive impacts, a generational reproductive study was conducted. Sperm parameters were determined by CASA and epigenetic enzyme mRNA expression was determined by pathway-focused RT-PCR. Mice ambiently exposed to ADBAC+DDAC exhibited decreases in reproductive indices that persisted through the F1 generation. Male mice (F0) dosed with 120 mg/kg/day of ADBAC+DDAC exhibited decreased sperm concentration and motility that persisted through the F1 generation. Changes in the mRNA expression of chromatin-modifying enzymes in the testes were seen. Two histone acetyltransferases (Hat1 and Kat2b) were upregulated, and one lysine-specific demethylase (Kdm6b) was downregulated in the F0 generation. The DNA methyltransferase Dnmt1 was downregulated in F1 males. These changes in chromatin-modifying enzymes are known to decrease fertility and could be a mechanism for ADBAC+DDAC reproductive toxicity. In all experiments, the F2 generation was similar to the controls, showing multi-generational but not trans-generational epigenetic inheritance.

## 1. Introduction

Quaternary ammonium compounds (QACs) are antimicrobial agents that have a wide spectrum of biocidal activity and are effective against many bacteria, viruses, fungi, and protozoa. Ubiquitous use of QAC cleaning solutions in residential, commercial, and medical settings has resulted in widespread human exposure [1,2,3]. Alkyl dimethyl benzyl ammonium chloride (ADBAC, also called benzalkonium chloride or BAC) and didecyl dimethyl ammonium chloride (DDAC) are among the most common QACs utilized in household cleaners. In a study conducted prior to the COVID-19 pandemic, 80% of individuals had ADBAC and DDAC residues in their blood [2]. A second study evaluating fifteen different QAC species in human blood samples before and during the pandemic found a significant increase of 77% in the median concentrations of total QACs, and an increase of 174% in the median concentration of total ADBAC species [3].

Previously, we reported declines in breeding performance and reproductive outcomes in mice exposed to ADBAC+DDAC [4,5,6]. ADBAC+DDAC exposure reduced breeding capacity in females through disturbances in estrus cyclicity, ovulation, and implantation, and reduced sperm concentration and motility in males [5]. ADBAC+DDAC exposure altered Sertoli cell cycling with a G2/M cycle arrest and significantly decreased Sertoli cell metabolism before disruption of the blood–testis barrier, showing a direct effect on Sertoli cell homeostasis [6].

Infertility is a significant clinical problem, affecting 8–12% of couples worldwide [7]. Approximately 40–50% of infertility cases are due to “male factor” infertility [8]. This includes a well-documented decline in sperm concentration from 113 million to 49.9 million sperm per mL [9,10]. According to World Health Organization guidelines, the assessment of sperm count is central to understanding male fertility [11].

Exposure to environmental pollutants can induce epigenetic changes in gene expression [12]. DNA modifications and chromatin remodeling enzymes regulate DNA replication, repair, and transcription. The addition or removal of specific chemical groups to DNA or chromatin induces structural changes to the 3-dimensional architecture of chromatin that regulates access to the underlying genetic sequence. Epigenetic alterations are more likely than DNA sequence mutations to dominate phenotypes among progeny [13]. The consequences of aberrant epigenomic remodeling are well documented in numerous disorders, including cancer, diabetes, and schizophrenia [14,15,16,17,18]. Epigenomic remodeling also occurs with environmental chemical exposure [19]. Epigenetic changes to germ cells from toxicant exposure can result in declines in fertility and can be passed on to future generations [19,20]. Germ cells for the next generation of offspring (the F1 generation) are present in adults (the F0 generation), and are vulnerable to harmful effects from parental toxicant exposures. The observation of toxic effects in the F1 generation following exposure of the F0 parents is termed multi-generational transmission. If the F2 or future generations of offspring display toxic effects, it is termed trans-generational transmission [20].

We have previously shown that ADBAC+DDAC are developmental toxicants causing neural tube defects in mice [21]. Developmental toxicity suggested the involvement of epigenetic mechanisms. Fetal malformations showed trans-generational inheritance that persisted through the F2 generation. Additionally, male-only exposure was sufficient to induce malformations in the offspring of unexposed mothers, implying epigenetic changes to sperm DNA.

Considering the almost universal human exposure to QACs and their negative impact on reproduction, it is vital we have a better understanding of the mechanisms of QAC reproductive toxicity. The goal of the current study is to determine whether ADBAC+DDAC exposure affects reproductive performance for multiple generations to confirm if the reproductive toxicity of these compounds acts through epigenetic mechanisms. Specifically, we will ascertain reproductive indices and sperm characteristics for two generations following ADBAC+DDAC exposure. We will also study mRNA expression of chromatin-modifying enzymes to assess epigenetic changes for two generations following ADBAC+DDAC exposure. We found multi-generational transmission but not trans-generational transmission for reproductive indices, sperm parameters, and the mRNA expression of chromatin-modifying enzymes.

## 2. Materials and Methods

### 2.1. Animal Husbandry for Mating Indices and Pregnancy Rates

**Figure 1:**
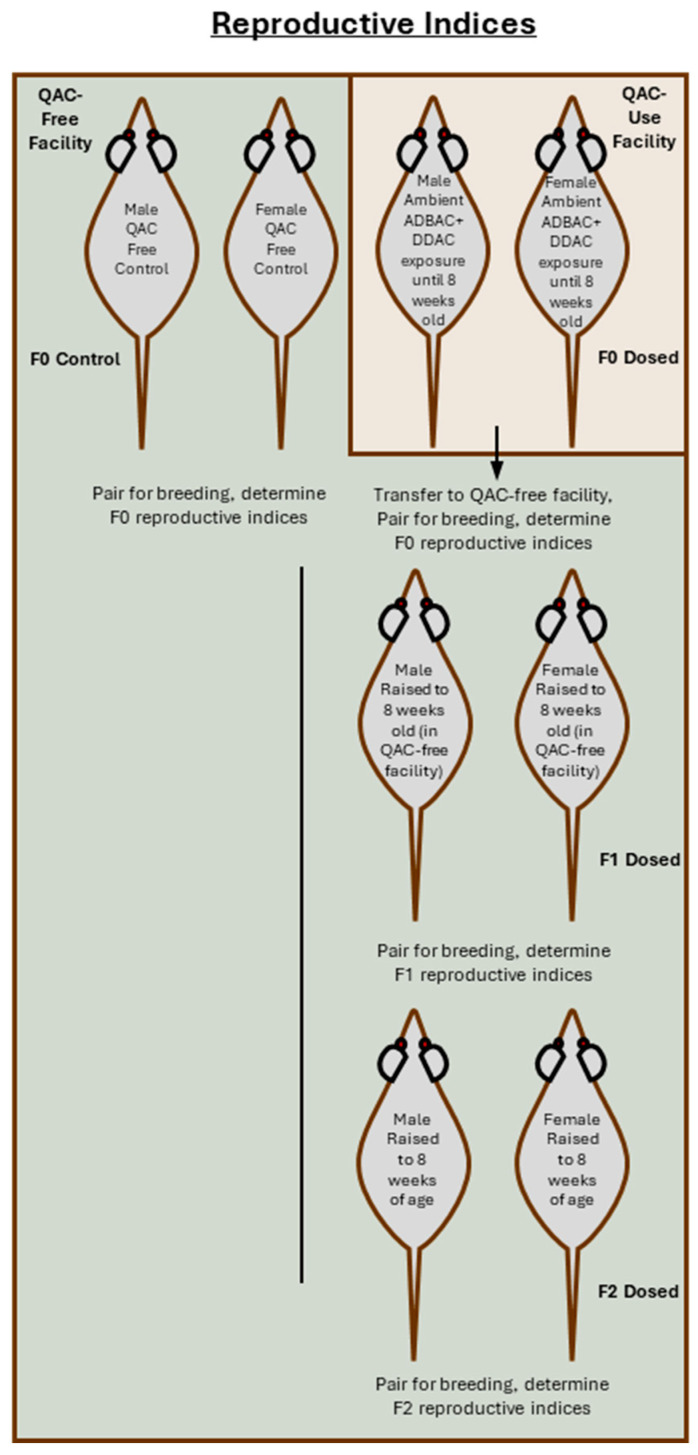
Schematic of experimental design for determination of reproductive indices. F0 mice were exposed ambiently in a QAC-use facility to normal use of an ADBAC+DDAC disinfectant in the mouse room while in-utero and until 8 weeks of age. Exposed mice were then transferred to the QAC-free facility for mating and gestation. At 8 weeks of age, F1 generation mice were bred to produce an F2 generation which was reared until 8 weeks of age before being bred to determine reproductive indices.

All animal experiments were approved by the Virginia Tech IACUC and conducted at the VA-MD College of Veterinary Medicine, Virginia Tech, an AAALAC accredited facility. CD-1 mice (*Mus musculus*) were obtained from Charles River Laboratories (Wilmington, MA, USA). The CD-1 mouse is a common outbred strain used in a broad range of biomedical studies, including toxicology and reproductive biology. As an outbred strain, its genetic diversity makes the CD-1 mouse a good model for the human population. Mice were housed in disposable caging (Innovive, San Diego, CA, USA) on a 12-h light/dark cycle at 20–25 °C with 30–60% relative humidity. Harlan 7013 rodent chow (Harlan Teklad, Madison, WI, USA) and distilled water were provided ad libitum. Mice were not dosed but were ambiently exposed to a QAC disinfectant with 6.76% ADBAC (60% C-14, 25% C-12, 15% C-16) and 10.1% DDAC (Sanitation Strategies, Holt, MI, USA) through routine husbandry cleaning and disinfection practices. These mice were bred to produce an F0 generation that was ambiently exposed in-utero to ADBAC+DDAC. At 8 weeks of age, the F0 mice were transferred to a QAC-free (QF) facility which used a chlorine dioxide disinfectant and paired (two females to one unrelated male) for breeding to derive an F1 generation. F1 mice were gestated and reared in the QAC-free facility until they were 8 weeks old before being paired for breeding (two females to one unrelated male) to derive an F2 generation. The F2 generation was reared and then bred at 8 weeks of age in the QAC-free facility (Figure 1). Reproductive indices were determined for each generation and compared to age matched unexposed controls bred and reared in the QAC-free facility.

### 2.2. Mating Indices and Pregnancy Rates

Mating indices and pregnancy rates were assessed in Control (N = 13), F0 (N = 8), F1 (N = 11), and F2 (N = 10) mated pairs for each generation. Mating index was calculated by assessing the number of females with copulatory plugs, divided by the number of females co-habited with a male. Pregnancy rate was calculated based on the number of females pregnant, divided by the number of females with evidence of breeding (i.e., presence of a copulatory plug).

### 2.3. Husbandry for Generational Assessment of Sperm Parameters and Testis mRNA Expression

**Figure 2:**
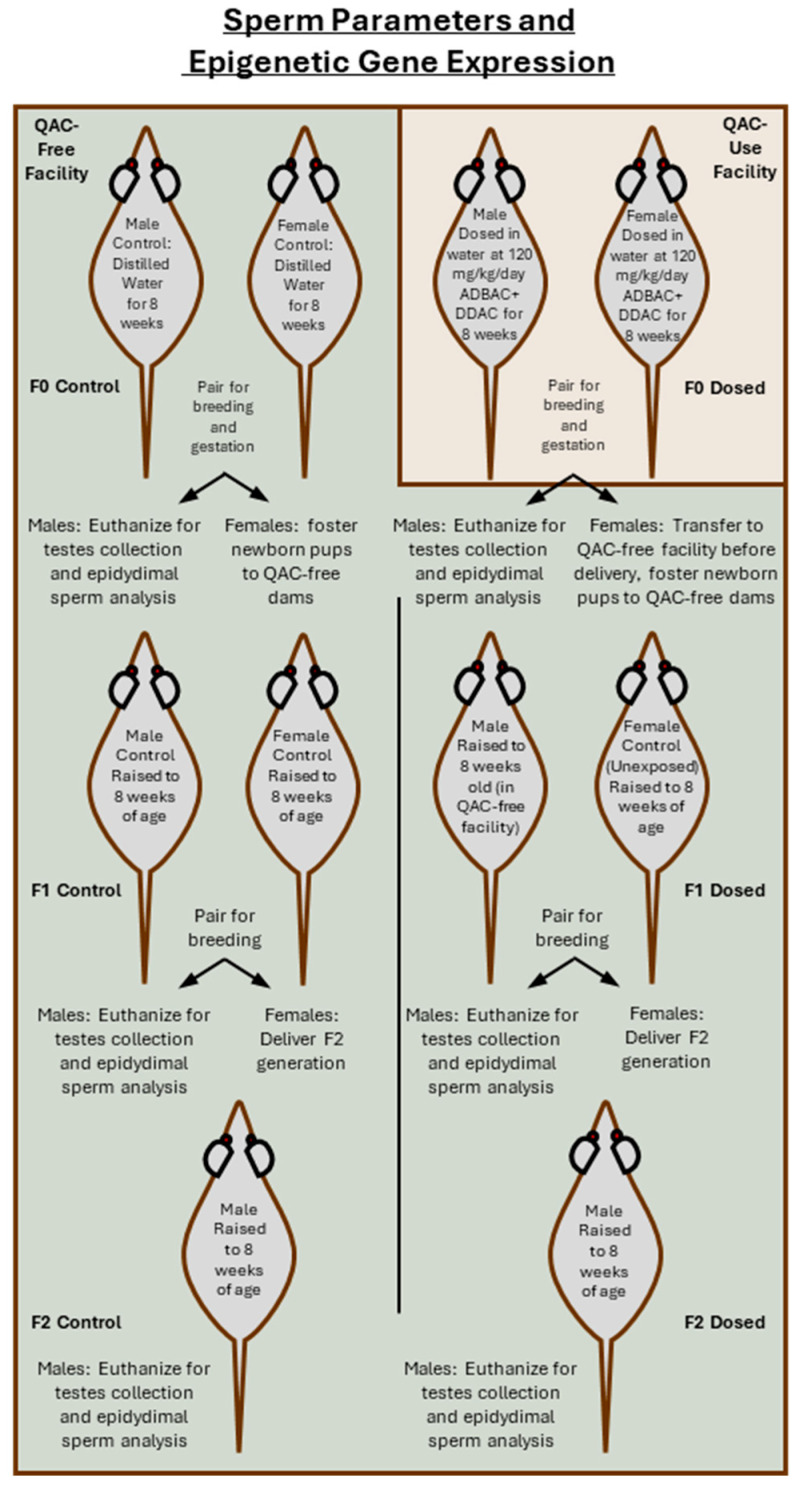
Schematic of experimental design for determination of sperm parameters and epigenetic gene expression. F0 male mice were dosed in the drinking water with 120 mg/kg/day ADBAC+DDAC for 8 weeks before breeding to unrelated F0 females also dosed at 120 mg/kg/day. Pregnant F0 females were transferred to the QAC-free facility the day before delivery. Newborn exposed and control F1 pups were cross fostered to unexposed control females at birth. Dosed F0 males were euthanized for testis collection and epididymal sperm analysis. The F1 generation was raised for 8 weeks in the QAC-free facility before being bred to unexposed control females to produce an F2 generation. F1 males not used for breeding were euthanized for testis collection and epididymal sperm analysis. F2 generation males were raised to 8 weeks of age before testis collection and epididymal sperm analysis.

A QAC free CD-1 mouse breeding colony was created as described previously [21]. Tissues from the control colony were checked periodically to ensure no contamination of the colony with QACs. Upon initiation of an experiment, dosed groups were transferred to a vivarium utilizing ADBAC+DDAC disinfectants. Control mice remained in the QAC-free facility. Housing mice in separate facilities was necessary to prevent contamination of the controls [4,21]. Mice were housed in disposable caging (Innovive, San Diego, CA, USA) on a 12-h light/dark cycle at 20–25 °C with 30–60% relative humidity. Harlan 7013 rodent chow (Harlan Teklad, Madison, WI, USA) and distilled water were provided ad libitum. Husbandry conditions, including the husbandry staff, were the same for both experiments and for both animal facilities. For the F0 exposure group, QAC-free mice were transferred after weaning at 3 weeks of age to the ADBAC+DDAC use facility (Figure 2). F0 male and female mice were dosed at 120 mg/kg/day by adding ADBAC+DDAC to distilled drinking water. The concentrations of ADBAC+DDAC were determined based on the sum of ADBAC+DDAC (6.76% ADBAC (60% C-14, 25% C-12, 15% C-16) and 10.1% DDAC), with an average daily water consumption of 10% body weight provided daily. Control mice and foster dams remained in the QAC-free facility and received distilled water. Water consumption was documented and was not significantly different between treatment groups. A dose of 120 mg/kg/day was chosen as it is close to the “no observed adverse effect level” or NOAL. During the study, no overt clinical signs of toxicity other than occasionally a very mild roughening of the hair coat was observed. No mortalities occurred during the study, and a daily general activity assessment found no difference in general activity and alertness between treatment groups. In the F0 generation, exposure to ADBAC+DDAC in the drinking water continued for 8 weeks and throughout breeding (one female paired with one unrelated male) and gestation. On gestational day 19, one day before delivery, exposed F0 females were transferred to the QAC-free facility for delivery. Exposed F0 males (N = 10) were euthanized by CO_2_ inhalation for testis and epididymis collection. Both control and dosed F1 progeny were cross fostered at birth to unexposed control dams. At 8 weeks of age, F1 males were bred to unexposed control females (one male paired with one unrelated female, N = 12 breeding pairs) to derive an F2 generation of males (N = 10). Testis and epididymis tissue was collected from F1 and F2 generation males for sperm and gene expression analysis (Figure 2).

### 2.4. Sperm Collection and Computer-Automated Sperm Analysis (CASA)

For sperm collection, both cauda epididymes were excised and placed in one mL of pre-warmed Dulbecco’s Modified Eagle’s Medium (DMEM) supplemented with 1% fetal bovine serum (GIBCO, Grand Island, NY, USA). Cauda epididymes were cut and incubated at 37 °C with 5% CO_2_ for 10–15 min to release sperm. A 500 µL aliquot of cauda epididymal extract was transferred to a 1.5 mL conical tube and diluted to 1.5 mL with pre-warmed DMEM. Sperm concentration and motility were evaluated in F0, (N = 10), F1 (N = 12), and F2 (N = 10) generation males. Duplicate 3 µL sample extracts were analyzed using 4-chamber Leja slides (Minitube of America, Verona, WI, USA). Slides were allowed to settle for 2–3 min before analysis. Eight random fields were analyzed with 20X phase-contrast at 30 frames per second (Model BX41, Olympus Optical, Tokyo, Japan) using Sperm Vision 3.5 (Minitube of America, Verona, WI, USA) to calculate sperm concentration and motility. Sperm following a non-linear, linear, or curvilinear swimming pattern were classified as motile, while non-swimming sperm were classified as immotile.

### 2.5. mRNA Expression Determination

The left and right testes (epididymis removed) from three unrelated control and three unrelated ADBAC+DDAC exposed males for each generation were used for generational assessment of ADBAC+DDAC on mRNA expression. Testis mRNA was isolated using an RNeasy Mini Kit according to manufacturer instructions (Qiagen, Hilden, Germany). The concentration and purity of mRNA was evaluated using a Nanodrop 2000 UV-Vis Spectrophotometer (Thermo Scientific, Waltham, MA, USA). Only mRNA with a 260/280 absorbance ratio ≥ 2.0 was used for RT-PCR analysis. Starting concentrations of mRNA were adjusted to 0.5 μg using nuclease-free water before cDNA synthesis using a RT^2^ First Strand Kit (SABiosciences, Frederick, MD, USA). RT^2^ SYBR Green qPCR Mastermix (SABiosciences, Frederick, MD, USA) was added to cDNA per manufacturer instructions. The Mouse Epigenetic Chromatin Modification Enzymes RT2 Profiler™ PCR Array (SABiosciences, Frederick, MD, USA) was used to detect the mRNA expression of enzymes known or predicted to modify genomic DNA and histone structure. The PCR Array configuration was a 96-well plate with primer assays for 84 pathway genes and 5 housekeeping genes to normalize array data. One well with a genomic DNA control, three wells with reverse-transcription controls, and three wells with positive PCR controls were used to evaluate inter-well/intra-plate accuracy and precision. PCR amplification was conducted using Bio-Rad iCYCLER iQ5 Real Time PCR instrument with the following cycling conditions: 1 cycle for 10 min at 95 °C, 40 cycles for 15 s at 95 °C, and 1 min at 60 °C. Cycle threshold was set at the point of detectable SYBR fluorescence.

### 2.6. Statistics

In all experiments, the number of animals (N) was based on the number of F0 individuals evaluated or the number of F1 or F2 litters evaluated, as litters are considered a subset of the originally exposed F0 individuals. Sperm parameter data were evaluated using Analysis of Variance (ANOVA) (Statistix, Tallahassee, FL, USA) with significance set at *p* ≤ 0.05. Data are expressed as the mean ± SEM. SA Biosciences RT^2^ PCR Data Analysis version 3.5 was used to generate the average Ct, 2^(−Ct)^, fold change, *p*-value, and fold regulation for each gene from RT-PCR Ct values. Fold changes were evaluated using T-tests to determine *p*-values. Changes in gene expression for exposed generations were compared to the generation matched controls. Genes with greater than 2-fold changes in expression and with *p* ≤ 0.05 were considered biologically and statistically significant.

## 3. Results

### 3.1. Mating Indices and Pregnancy Rates

**Figure 3:**
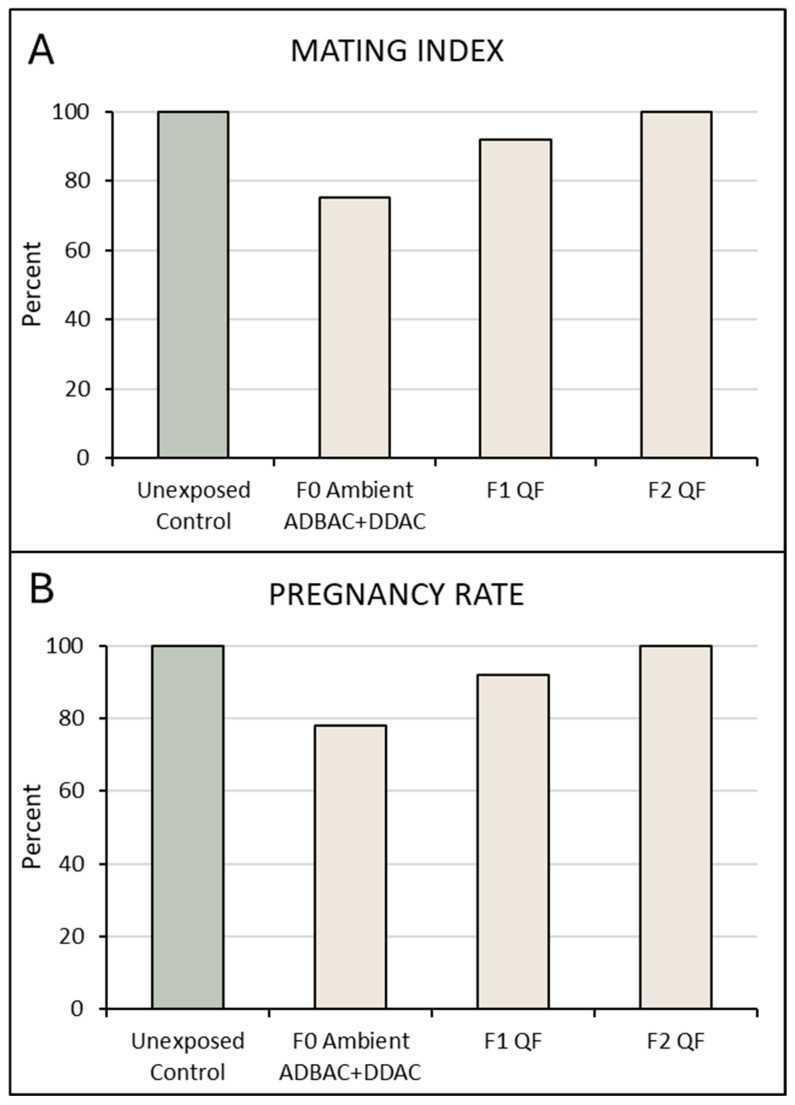
Mating indices (**A**) and pregnancy rates (**B**) following ADBAC+DDAC exposure. F0 females were ambiently exposed in-utero and for 8 weeks after birth to an ADBAC+DDAC disinfectant. Control mice, and the F1, and F2 progeny of the exposed F0 were gestated and raised in a QAC-free facility. Green bars represent controls while tan bars represent ADBAC+DDAC generations. Mating indices and pregnancy rates were calculated for each generation. Mating index: (number of females with copulatory plug/number of females cohabitated with a male) × 100; Pregnancy rate: (number of females who became pregnant/number of females with a copulatory plug) × 100. N = 8–11 litters per generation.

Mating index and pregnancy rate were calculated for the ambiently exposed F0 generation and for the following F1 and F2 generations bred and reared in the QAC-free facility. F0 mice, which were ambiently exposed in-utero and for 8 weeks after birth to an ADBAC+DDAC disinfectant, had a mating index and pregnancy rate of 75% and 78%, respectively (Figure 3). F1 mice, derived from the ambiently exposed F0 females but gestated and raised in the QAC-free facility, had both a mating index and pregnancy rate of 92%. The F2 generation (born and raised in the QAC-free facility) had both a mating index and a pregnancy rate of 100% (Figure 3). This demonstrates that the reduction in reproductive indices only persists into the F1 generation through direct exposure of gametes in F0 mice.

### 3.2. Sperm Concentration and Motility

**Figure 4:**
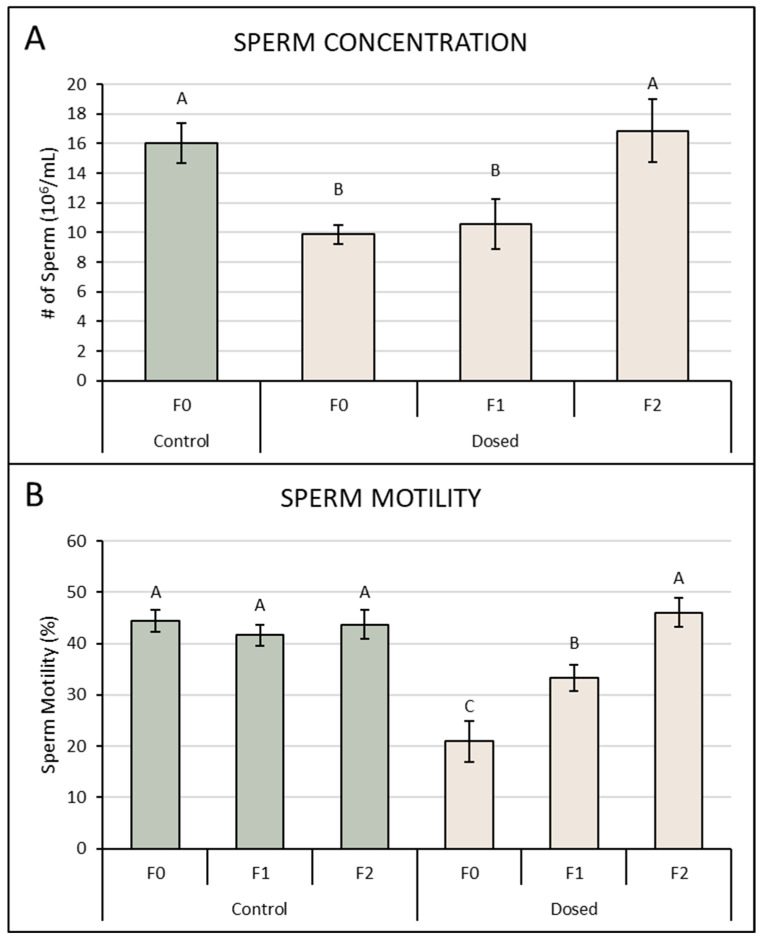
Sperm concentration and motility across generations in mice exposed in the F0 generation to ADBAC+DDAC at 120 mg/kg/day in the drinking water for 8 weeks and through breeding and gestation. Dosed F0 males were euthanized for testis collection and epididymal sperm analysis. Pregnant dosed F0 females were transferred to the QAC-free facility immediately prior to delivery. F1 generation pups were cross-fostered to unexposed control dams at birth. F1 generation males were reared to 8 weeks of age and were bred to age-matched unrelated control females to produce an F2 generation. Sperm metrics were determined in F2 males at 8 weeks of age. (**A**) Sperm concentration was significantly lower in directly exposed F0 males and in F1 males but returned to control concentrations in the F2 generation. (**B**) Sperm motility in F0 ADBAC+DDAC exposed males was decreased. Motility remained low in F1 males but returned to control levels in the F2 generation. Green bars represent controls while tan bars represent ADBAC+DDAC generations. N = 8–10 males/generation, Bars denote the mean ± SEM; bars with different letters are significantly different by ANOVA with *p* ≤ 0.05.

Exposure to 120 mg/kg/day ADBAC+DDAC for 8 weeks significantly reduced sperm parameters in F0 mice (Figure 4). Control F0 mice had sperm concentrations of 16.0 ± 1.3 × 10^6^ sperm/mL, compared to 9.9 ± 0.6 × 10^6^ sperm/mL in ADBAC+DDAC exposed F0 mice. Sperm concentration in F1 males increased to 10.6 ± 1.7 × 10^6^ sperm/mL. Sperm concentration increased again in the F2 generation to 16.8 ± 2.1 × 10^6^ (Figure 4). Sperm motility was significantly decreased in directly exposed F0 males with 20.9 ± 4.1% motile sperm compared to 44.4 ± 2.5% motile sperm in control males (Figure 4). Sperm motility increased for F1 males to 33.3 ± 2.8%. Sperm motility in F2 males that were gestated and reared in the QAC-free facility increased to 46 ± 2.9% (Figure 4). Restoration of sperm parameters to control levels in the F2 generation denotes multi-generational but not trans-generational heritable changes to sperm.

### 3.3. Gene Expression of Chromatin-Modifying Enzymes

**Figure 5:**
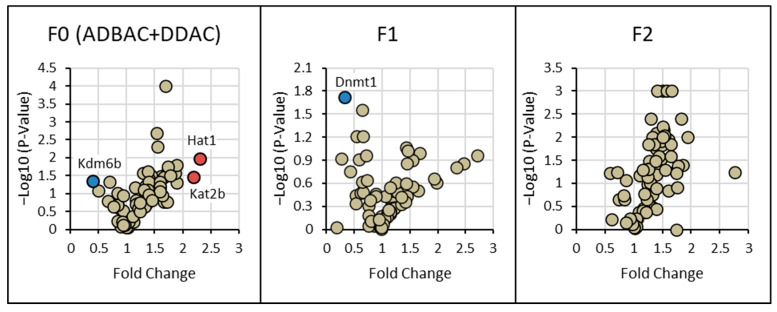
Volcano plots showing mRNA expression of chromatin-modifying enzymes in the testes of F0 directly exposed to ADBAC+DDAC and subsequent F1 and F2 generation males compared to age- and generation-matched controls. Significant changes were seen in F0 males that were directly exposed to 120 mg/kg/day ADBAC+DDAC in the drinking water for 8 weeks. In F0 exposed males, the two histone acetyltransferases, Hat1 and Kat2b, were upregulated, while one histone, demethylase Kdm6b, was downregulated. F1 progeny had significant downregulation of Dnmt1, a DNA methyltransferase. No significant changes were seen in F2 males. Fold changes greater than 2 with *p* ≤ 0.05 were considered biologically and statistically significant.

A complete list of results for the expression of all chromatin-modifying enzymes is given in Supplemental Table S1. The highest frequency of significant fold changes in mRNA expression compared to controls was seen in F0 males exposed to ADBAC+DDAC (Figure 5). F0 ADBAC+DDAC-exposed males had upregulation of Hat1 and Kat2b histone acetyltransferases and downregulation of Kdm6b histone demethylase in testicular tissue. In F1 ADBAC+DDAC mice only exposed in-utero, the only gene that was differentially expressed from controls was downregulation of Dnmt1 DNA methyltransferase. This suggests that in-utero exposure to ADBAC+DDAC induced post-natal changes to the testicular epigenome. There were no significant differences in testicular gene expression between the ADBAC+DDAC F2 generation and the control F2 generation. This suggests that exposure to ADBAC+DDAC induces multi-generational epigenetic changes but not trans-generational changes.

## 4. Discussion

ADBAC and DDAC are often found together in consumer and industrial products. Studies evaluating toxicity usually evaluate ADBAC and DDAC separately despite humans being exposed to mixtures of QACs simultaneously. Toxicity from mixtures can manifest differently than toxicity from individual compounds. Only a few studies from our lab have evaluated the combined effects of ADBAC+DDAC in-vivo [2,4,5,6,21]. Toxicity to a variety of QACs in consumer products is well documented, including corneal cytotoxicity, allergic rhinitis, contact dermatitis, and occupational asthma [22,23,24,25,26,27]. Studies evaluating systemic toxicity have shown that QACs alter sterol and lipid homeostasis and inhibit mitochondrial function in cell culture, rodents, and in humans [2,28,29,30]. Nevertheless, QACs remain prevalent in consumer and industrial products and are viewed as safe; however, this paradigm is shifting as more studies are finding significant toxicity associated with QAC exposure [31].

Epigenetic modifications induce changes in gene expression without changing the underlying DNA sequence. Epigenetic modifications include DNA methylation, histone modification, and non-coding mRNA (ncRNA)-associated gene silencing [32]. DNA methylation usually occurs on cytosines of CpG sequences. Methylation of CpG sequences in promotor regions results in gene silencing. Inactive genes are typically hypermethylated while transcriptionally active genes are hypomethylated. Histone modification occurs through addition or removal of typically a methyl, acetyl, or phosphate group. Histone modification can enhance gene expression by relaxing DNA to enable transcription or condensing DNA to silence genes [32]. Histone acetylation typically induces transcriptional upregulation through chromatin relaxation [33]. Histone methylation can either enhance or reduce gene transcription depending on the specific circumstances [32].

**Figure 6:**
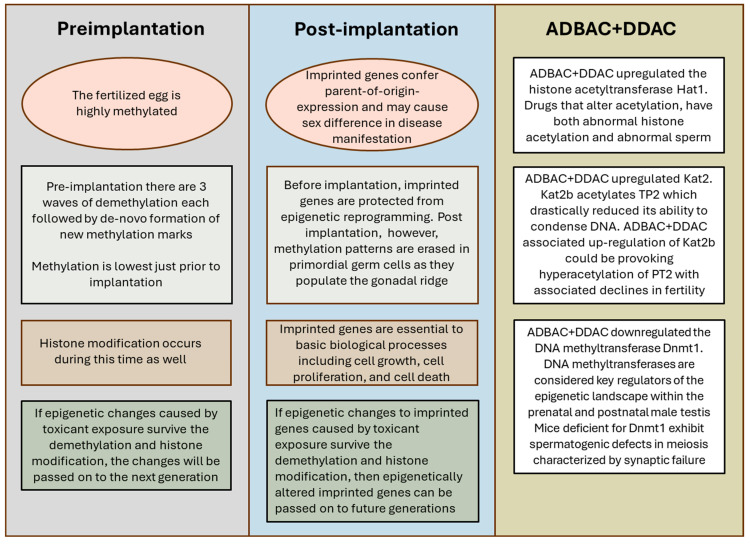
Schematic summarizing major events occurring during epigenetic reprogramming and how these events can result in transmission of epigenetic changes to future generations. Also summarized is the effect of ADBAC+DDAC exposure on chromatin-modifying enzymes and their possible impact on reproduction.

Epigenetic changes are transmissible through gametes and, therefore, between several generations. The main mechanism for transmission of epigenetic determinants across generations is epigenetic reprogramming (Summarized in Figure 6). As reviewed by Klibaner-Schiff et al. [19], both the egg and sperm DNA are highly methylated. Post fertilization, there are three successive waves of demethylation which allow the zygote to become pluripotent. Methylation of DNA is at its lowest shortly before implantation. Following each wave of demethylation, there is de novo formation of new methylation marks. Histone modification occurs as well. If epigenetic changes to egg and sperm DNA from toxicant exposure survive the demethylation and histone modification events, then the changes from toxicant exposure can be passed on to future generations [19]. For example, the agricultural herbicide vinclozolin is a known anti-androgenic endocrine disruptor. Vinclozolin altered sperm concentrations and methylation status of paternally derived H19 and Gtl2 and maternally derived Peg1, Snrpn, and Peg3 in the sperm of F1 offspring [34]. The changes persisted trans-generationally but dissipated with each following generation. Decreased spermatogenesis and infertility were also seen in adult mice gestationally exposed to the estrogenic insecticide methoxychlor. These reproductive deficiencies persisted into the F2 generation and were not correlated with tissue abnormalities or altered serum testosterone concentrations [35].

Germ-line specific patterns of DNA methylation are formed by methyltransferases. The germ-line specific methylation pattern is sex-specific and is formed during gonadal differentiation [36]. Primordial germ cells are directed by these methylated loci to develop into oocytes or sperm, depending on the sex of the embryo. In-utero exposure to toxicants that interfere with the creation of methylation patterns during gonadal differentiation cause heritable alterations in cell differentiation, proliferation, and, ultimately, function [19,37,38,39]. In-utero exposure to endocrine disrupting compounds (EDCs) during gonadal differentiation can interfere with germ cell DNA methylation, resulting in male subfertility that persists for several generations [38].

In the present study, ADBAC+DDAC exposure upregulated the histone acetyltransferase (Hats) Hat1 expression in testes. Hats transfer an acetyl group derived from acetyl-coA to an exposed lysine on histone tails. Regulation of histone acetylation is critical for spermatogenesis. As reviewed by Ou et al. [33], a number of studies have shown that mice genetically knocked out or treated with drugs to alter acetylation have both abnormal histone acetylation and abnormal sperm. Diminished sperm parameters in F0 ADBAC+DDAC exposed males coincided with upregulation of Hat1, which could be causing hyperacetylation of histones and declines in sperm number and quality.

F0 males exposed to ADBAC+DDAC also showed significant upregulation of Kat2b, which is involved in spermiogenesis. Once spermatogonia are formed during spermatogenesis, they undergo a maturation process called spermiogenesis. During spermiogenesis, chromatin is remodeled; nucleosomal histones are replaced by transition proteins TP1, TP2, and TP4, which are then replaced by protamines [40]. Both histone hyperacetylation and replacement of histones by protamines relax the nucleosome [33]. P2 aids in replacing histones with protamines during spermiogenesis and binds to and condenses DNA [41]. Kat2b acetylates TP2, which drastically reduces its DNA condensation ability [40]. ADBAC+DDAC-associated upregulation of Kat2b in F0 exposed males could be provoking hyperacetylation of PT2, with associated declines in fertility.

Kdm6b is a lysine-specific histone demethylase whose expression is primarily localized to undifferentiated spermatogonia. Downregulation of Kdm6b expression is associated with the destabilization of germ-cell intercellular bridges [42]. In contrast, male mice enriched with Kdm6b demonstrate larger testicles and stay fertile longer than their unmodified counterparts [42]. This suggests a significant role for Kdm6b in spermatogonial proliferation and differentiation. In the present study, Kdm6b was downregulated in the testes of ADBAC+DDAC-exposed F0 male mice. Reductions in spermatogenic efficiency due to insufficient Kdm6b expression may be a mechanism by which ADBAC+DDAC exposure reduces sperm parameters.

In the testes of F1 males exposed to ADBAC+DDAC in-utero, the DNA methyltransferase Dnmt1 was significantly downregulated. DNA methyltransferases are considered key regulators of the epigenetic landscape within the prenatal and postnatal male testis [43]. Mice with altered Dnmt1 expression show abnormal methylation patterns in spermatogenic populations when compared to controls [44]. Dnmt1 maintains DNA methylation patterns during spermatogenesis by forming complexes with the SRA protein Np95. Np95 facilitates the loading of Dnmt1 to replicating heterochromatic regions of DNA [45]. Mice deficient for Np95 or Dnmt1 exhibit spermatogenic defects in meiosis characterized by synaptic failure [46]. From their findings, Takada et al. [46] propose that DNA methylation patterns formed in pre-meiotic spermatogonia control the synapsis of homologous chromosomes, which in turn contributes to the quality control of male germ cells and the production of mature sperm. Dnmt1 downregulation coincided with reduced sperm parameters in the F1 generation, possibly indicating another mechanism for impaired male reproduction seen with ADBAC+DDAC exposure.

Of particular concern is the possibility that QAC exposure can make epigenetic changes to imprinted genes (summarized in Figure 6). Imprinted genes have methylation marks that confer parent-of-origin-specific expression, and may cause sex difference in disease manifestation. Imprinted genes are established in gametes during early gonadal development in the embryo and, therefore, can reflect environmental exposures of the preceding generation [47]. Imprinted genes are susceptible to epigenetic modifications [34,48]. After fertilization, imprinted genes are protected from epigenetic reprogramming occurring prior to implantation; however, methylation patterns are erased in the post-implantation embryo. As primordial germ cells are specified and migrate into the gonadal ridge in the early embryo, there is loss of histone modification and methylation to reactivate imprinted genes [19]. If toxicant-induced epigenetic changes to imprinted genes survive the demethylation and histone modification events, then epigenetically altered imprinted genes can be passed on to future generations [49]. This is of concern as imprinted genes are fundamental to many basic biological processes, including embryonic, organismal, and cellular development, as well as cell growth, cell proliferation, and cell death. A number of disease states, including gastrointestinal disease, endocrine system disorders, hereditary disorders, various cancers, disorders of growth and metabolism, and disorders in neurodevelopment, cognition, and behavior, including certain major psychiatric disorders, are linked to imprinted genes [48,50].

## 5. Conclusions

This study supports the earlier findings that ADBAC and DDAC are reproductive toxicants in mice, reducing both male and female reproductive capabilities in a multi-generational manner. Ambient exposure of the F0 generation reduced reproductive indices in the F0 and F1 generations. Dosing with ADBAC+DDAC at 120 mg/kg/day in the drinking water reduced sperm counts and motility in the F0 and F1 generations. Dosing with ADBAC+DDAC at 120 mg/kg/day induced changes in key epigenetic enzymes associated with reduced fertility and abnormal sperm development and maturation in the F0 and F1 generations. In all experiments, the F2 generation was equivalent to the controls, signifying no trans-generational effects from exposure. We have previously shown that ADBAC+DDAC affects Sertoli cell homeostasis and function [6]. The results from the present study show toxic effects, particularly epigenetic changes, likely occur in developing sperm as well. While this study identified possible mechanistic pathways for impaired reproduction, more work is needed to identify the exact mechanisms and biochemical pathways involved. Future studies should evaluate the effect of ADBAC+DDAC exposure on each altered gene to gain a better understanding of how ADBAC+DDAC impacts reproduction.

Our previous results showing reproductive and developmental toxicity associated with ADBAC+DDAC exposure and the current findings of multi-generational reproductive and epigenetic effects in mice highlight the importance of clarifying the toxicity of these compounds in the human population. Human exposure to products having a variety of QACs as ingredients is frequent and ubiquitous. Products such as cleaners, personal care and hair products, laundry products, air fresheners, and many others that people encounter everyday have QACs. There are over 200 species of QACs, and toxicity has only been evaluated for a few. It is not known whether ADBAC+DDAC exposure in humans is linked to specific disease states other than allergic rhinitis, contact dermatitis, and occupational asthma. However, the ability to cause multi-generational epigenetic changes, impair mitochondrial function, alter sterol formation and homeostasis, and function as an EDC all highlight the possibility that QACs may have profound effects on human health and disease.

## Data Availability

The original contributions presented in this study are included in the article/Supplementary Material. Further inquiries can be directed to the corresponding author(s).

## Supplementary Materials

The following supporting information can be downloaded at: https://www.mdpi.com/article/10.3390/toxics13090709/s1, Supplemental Table S1. Changes in testis mRNA expression of chromatin remodeling enzymes in F0 males dosed for 8 weeks to 120 mg/kg/day ADBAC+DDAC in the drinking water and their unexposed F1 and F2 progeny. The pathway focused Mouse Epigenetic Chromatin Modification Enzymes RT2 profiler™ PCR Array (SABiosciences, Frederick, MD, USA) was used to identify changes in epigenetic gene expression.

## Abbreviations

The following abbreviations are used in this manuscript:

QACs	Quaternary Ammonium Compounds
ADBAC	Alkyl Dimethyl Benzyl Ammonium Chloride
DDAC	Didecyl Dimethyl Ammonium Chloride
DMEM	Dulbecco’s Modified Eagle’s Medium
CASA	Computer-automated Sperm Analysis
Hats	Histone Acetyltransferase
EDCs	Endocrine Disrupting Compounds
