# Supplementary material for: Direct and In-Utero Exposure to Quaternary Ammonium Disinfectants Alters Sperm Parameters and mRNA Expression of Epigenetic Enzymes in the Testes of Male CD-1 Mice
Source: Toxics. 2025 Aug 23;13(9):709. doi: 10.3390/toxics13090709 (PMC12473625; doi:10.3390/toxics13090709)
Supplement: Supplementary file 1 [file toxics-13-00709-s001.zip › toxics-3748495-supplementary.pdf]

Supplementary material.

Direct and In-utero Exposure to Quaternary Ammonium Disinfectants Alters Sperm Parameters and mRNA Expression of Epigenetic Enzymes in the Testes of Male Mice

Table S1

mRNA Expression of Chromatin Modifying Enzymes<br>Fold change compared to generation and age matched controls														
Gene	F0 ADBAC+DDAC		F1 Unexposed		F2 Unexposed		Gene	F0 ADBAC+DDAC		F1 Unexposed		F2 Unexposed	
	Fold change	p-value	Fold change	p-value	Fold change	p-value		Fold change	p-value	Fold change	p-value	Fold change	p-value
Ash1l	1.55	0.005	0.64	0.028	1.52	0.007	Mysm1	1.78	0.031	1.14	0.585	1.64	0.026
Atf2	1.67	0.169	0.54	0.062	1.82	0.004	Kat8	1.29	0.027	2.71	0.108	1.33	0.010
Aurka	1.36	0.096	1.06	0.647	1.17	0.429	Kat7	1.38	0.024	1.34	0.468	1.1	0.068
Aurkb	1.36	0.136	1.14	0.622	1.31	0.318	Kat6a	1.57	0.105	0.9	0.767	1.49	0.015
Aurkc	1.72	0.167	0.85	0.891	2.76	0.057	Kat6b	1.49	0.100	0.18	0.937	1.48	0.012
Carm1	1.24	0.165	0.53	0.358	1.37	0.016	Ncoa1	1.12	0.603	0.76	0.490	1.05	0.855
Cdyl	1.61	0.033	0.61	0.335	1.22	0.071	Ncoa3	1.57	0.078	0.26	0.121	1.55	0.051
Csrp2bp	0.86	0.260	0.42	0.177	0.87	0.085	Ncoa6	0.9	0.848	0.9	0.788	0.74	0.226
Dnmt1	1.89	0.050	0.33	0.019	1.02	0.927	Nek6	1.63	0.113	1.12	0.608	1.94	0.010
Dnmt3a	0.69	0.046	0.52	0.462	1.02	0.787	Nsd1	1.04	0.606	1.09	0.637	1.57	0.001
Dot1l	0.93	0.303	1.27	0.330	1.06	0.595	Pak1	1.31	0.177	1.55	0.279	1.24	0.104
Dzip3	1.7	0.000	1.17	0.449	1.5	0.001	Prmt1	0.97	0.750	2.34	0.157	1	0.896
Ehmt1	1.74	0.022	0.89	0.347	1.86	0.041	Prmt2	0.83	0.207	1.98	0.245	0.98	0.743
Ehmt2	1.16	0.155	1.67	0.101	1.6	0.143	Prmt3	1.4	0.047	1.93	0.221	1.74	0.059
Esco1	1.87	0.027	1.14	0.533	1.74	0.000	Prmt5	0.84	0.569	1.45	0.366	0.84	0.224
Esco2	1.47	0.041	1.06	0.370	1.4	0.018	Prmt6	0.5	0.081	1.42	0.433	0.71	0.058
Hat1	2.31	0.011	0.65	0.331	1.39	0.040	Prmt7	1.59	0.069	1.43	0.254	1.28	0.098
Hdac1	1.4	0.090	1.43	0.086	1.41	0.001	Rnf20	1.42	0.099	1.15	0.594	1.44	0.026
Hdac10	1.02	0.896	0.64	0.238	1.28	0.324	Rps6ka3	1.54	0.086	1.35	0.382	1.5	0.006
Hdac11	1.47	0.057	1.07	0.657	1.4	0.065	Rps6ka5	1.59	0.048	1.08	0.682	1.64	0.014
Hdac2	1.67	0.035	0.58	0.124	1.28	0.101	Setd1a	1.35	0.195	0.75	0.659	1.23	0.226
Hdac3	1.15	0.066	0.76	0.907	0.61	0.611	Setd1b	1.19	0.186	0.94	0.869	1.3	0.004
Hdac4	0.99	0.869	1.21	0.524	1.32	0.178	Setd2	1.6	0.063	0.8	0.785	1.35	0.014
Hdac5	1.33	0.068	2.47	0.137	1.73	0.055	Setd3	0.92	0.602	0.95	0.798	0.83	0.180
Hdac6	1.89	0.016	1.24	0.249	1.77	0.042	Setd4	1.38	0.080	1.44	0.138	1.54	0.009
Hdac7	1.5	0.103	0.95	0.927	1.17	0.346	Setd5	0.94	0.756	0.98	0.831	1.18	0.169
Hdac9	1.53	0.002	1.03	0.683	1.76	0.124	Setd7	0.77	0.237	0.97	0.951	0.98	0.772
Kat2a	1.43	0.031	1.03	0.767	1.27	0.033	Setd8	1.59	0.088	1.15	0.281	1.5	0.010
Kat2b	2.2	0.036	1.22	0.521	1.48	0.029	Setdb1	1.31	0.079	1.04	0.725	1.24	0.073
Kat5	1.5	0.096	1.54	0.127	1.09	0.432	Setdb2	1.11	0.419	1.12	0.550	1.24	0.049
Kdm1a	0.84	0.201	1.21	0.524	1.16	0.386	Smyd1	1.16	0.194	0.99	0.997	1.25	0.050
Kdm4a	0.67	0.163	0.72	0.231	1.39	0.355	Smyd3	1.21	0.255	1.01	0.857	1.4	0.129
Kdm4c	1.42	0.053	0.65	0.061	1.22	0.424	Suv39h1	0.93	0.113	0.99	0.940	1.16	0.052
Kdm5b	1.74	0.018	1.11	0.620	1.47	0.011	Suv420h1	1.32	0.235	0.71	0.110	1.38	0.033
Kdm5c	0.83	0.096	1.64	0.313	1.05	0.585	Usp16	1.4	0.107	0.88	0.371	1.26	0.014
Kdm6b	0.4	0.047	1.4	0.389	0.59	0.061	Usp21	1.23	0.314	0.78	0.420	0.93	0.595
Kmt2c	1.33	0.061	1.01	0.691	1.6	0.011	Usp22	1.45	0.156	1.46	0.095	1.66	0.001
